# Exploration of the Mechanism of the Control of Coccidiosis in Chickens Based on Network Pharmacology and Molecular Docking With the Addition of Modified Gegen Qinlian Decoction

**DOI:** 10.3389/fvets.2022.849518

**Published:** 2022-03-17

**Authors:** Xiaomin Peng, Kaijun Wang, Yuhan Wang, Yujie Lu, Feifei Lv, Yao Cui, Ying Wang, Hongbin Si

**Affiliations:** State Key Laboratory for Conservation and Utilization of Subtropical Agro-bioresources, College of Animal Science and Technology, Guangxi University, Nanning, China

**Keywords:** chicken coccidiosis, network pharmacology, molecular docking, mechanism of action, Modified Gegen Qinlian Decoction

## Abstract

Gegen Qinlian Decoction is a long-established Chinese herbal compound for the treatment of diarrhea and dysentery, while Magnolia officinalis has been demonstrated to have some anthelmintic activity. The preliminary screening of this study showed that the addition of Modified Gegen Qinlian Decoction has some effective on the prevention and treatment of coccidiosis in chickens. However, the mechanism of its treatment of chicken coccidiosis is not clear. The network pharmacology study was based on the screening of chemical components and related targets from TCMSP and PharmMapper server databases. Genes related to chicken coccidiosis were obtained from the SRA database, and those genes that intersected with the target genes of Modified Gegen Qinlian Decoction were screened. By exploring the target interactions through the String system and enrichment analysis by the Metascape system, the mechanism of action of Modified Gegen Qinlian Decoction in chicken coccidiosis was identified. Using real-time quantitative polymerase chain reaction (RT-qPCR) to analyze the mRNA levels of the relevant factors in chicken coccidiosis, molecular docking was used to reveal the extent of binding of the key target genes predicted in the network pharmacology by the action of Modified Gegen Qinlian Decoction. Compound and target screening suggested that the 99 chemical targets of Modified Gegen Qinlian Decoction were involved in chicken coccidiosis, and the enrichment results of KEGG pathway suggested that Modified Gegen Qinlian Decoction was significantly associated with PI3K/AKT signaling pathway in chicken coccidiosis. The Hubba gene module in Cytoscape_v3.7.1 software was used to analyze the network topology to obtain the Hubba gene SRC, STAT3, and PPARG, etc. The molecular docking results showed that SRC, STAT3, and PPARG were key targets in the treatment of coccidiosis in chickens by Modified Gegen Qinlian Decoction, which was in agreement with the RT-qPCR results. Through network pharmacology, molecular docking and *in vitro* experiments, it was confirmed that Modified Gegen Qinlian Decoction fights against chicken coccidiosis through key targets such as SRC, STAT3, and PPARG.

## Introduction

Chicken coccidiosis is one of the most common parasitic diseases in modern intensive poultry farming, caused by intracellular parasitic protozoa of the genus *Eimeria* (1). *Eimeria* is roughly divided into seven species from whether it is pathogenic, of which *Eimeria tenella* is the most common in farming situations. It is mainly parasitic in the intestinal tissues of birds, by attacking the cecum tissues of poultry animals, to affect their growth performance in serious cases directly to death. In January 2020, the implementation of the Chinese policy to reduce the use of veterinary antibiotics, Chinese medicine and its extracts to prevent and control chicken coccidiosis became one of the hot spots of market attention. Chicken coccidiosis typically results in impaired weight growth, reduced feed conversion, and increased mortality in diseased or infected chickens, and can cause economic losses of up to ~ £10.4 billion annually worldwide (2). Currently, chicken coccidiosis is primarily controlled by drugs and vaccines (3), however, due to certain safety risks and high costs of vaccine control, drug control is still the main means of prevention. With the emergence of drug-resistant strains and the increase in the cost of research on new anticoccidial drugs, the need for purely western drugs for prevention and control has not been met, and some western drugs also have the disadvantages of high toxic side effects and high drug residues (4). In China, herbal medicine resources are abundant, and from the current research status of herbal medicine developed for the control of coccidiosis, herbal medicine is mostly used for the control of coccidiosis in chickens and rabbits (5). The use of Chinese herbal medicines or the composition of compound drugs according to the combination of prevention and treatment of coccidiosis has unique advantages in anti-coccidial effect (6), enhancing immunity, antibacterial and antibacterial, alleviating clinical symptoms, etc. Moreover, Chinese herbal medicines have the advantages of wide drug source (7), cheap and easy to obtain, low toxicity, wide anti-worm spectrum, and not easy to produce drug resistance (5).

Traditional Chinese Medicine (TCM) has a long history, having been practiced in China for over 2000 years, and many herbal formulas have been passed down and accumulated through years of clinical experience (8). One of them is Gegen Qinlian Decoction(GQD), which is used to treat diarrhea and dysentery, GQD consists of four botanicals, namely, Pueraria Ohwi (Ge gen in Chinese, GG), Scutellaria baicalensis (Huang Qin in Chinese, HQ), Huang Lian (in Chinese HL), and Glycyrrhiza uralensis Fisch (Gan Cao in Chinese, GC) (9), the main bioactive substances of GQD were identified by 2D-LC/MS method, among which the most abundant components in Pueraria Ohwi, Scutellaria baicalensis, Huang Lian, and Glycyrrhiza uralensis Fisch were Puerarin, Baicalein, Berberine and Glycyyhizic acid, respectively (10). GQD achieves protection of the colonic mucosa in acute/chronic ulcerative colitis by modulating dysregulated Notch signaling; lowering glucose, regulating the intestinal microbiota, and relieving diabetic symptoms (11, 12). A grouping of GQD's main bioactive substances improves the disease of UC by altering the gut microbiota dependent (13). Magnolia officinalis Rehder & E. Wilson (Houpo) has been an important traditional Chinese medicine (TCM) for bloating, indigestion, and asthma treatment of coughs for centuries, including anti-inflammatory, anti-cancer, anti-epileptic, anti-depressant, anti-bacterial as well as anti-worm activity (14). Mangnolia officinalis var. biloba have been shown to have antihyperglycemic, cardioprotective, antioxidant, and antitumor activities (15). Magnolol may exert antifungal effects by blocking the oxidative phosphorylation metabolic pathway. Therefore, Magnolol was selected as the main drug, and Puerarin, Baicalein, Berberine, and Glycyyhizic acid were used to form the addition of Modified Gegen Qinlian Decoction (MGQD).

Chinese herbal remedies are more effective in treating the symptoms, but the complex composition of Chinese herbal medicines, with multi-component, multi-target and multi-potency characteristics, clearly elucidating the mechanism of action of how herbal remedies treat diseases are a challenging challenge. With the validation of a large number of experiments, more and more targets have been validated, and a series of databases containing targets corresponding to drug components and disease targets have been derived. Network pharmacology is such a novel approach, which uses the global prediction of the corresponding targets of drug components and disease targets to construct network models to predict the targets of action, screen the possible functions, and pathways of drugs to treat diseases, and reveal the potential mechanisms of action of drugs to treat diseases (16).

In this study, the differentially expressed genes (DEGs) detected under network pharmacology and RNA-Seq technology were used to explore the protective effect of MGQD active ingredient for the treatment of chicken coccidiosis and to further predict the therapeutic mechanism of MGQD active ingredient for chicken coccidiosis by pathway enrichment analysis of intersecting targets. To the best of our knowledge, this study is the first to explore the efficacy and mechanism of the effective active ingredient of MGQD against chicken coccidiosis, providing theoretical support and direction for further basic research.

## Materials and Methods

### Animal Experiments

Two hundred 1-day-old male broilers were randomly assigned to 12 sterile cages in an environmental *Eimeria*-free room and fed a basal diet as well as a supplemental diet that did not contain antibiotics or anticoccidials. Chickens had free access to feed and water throughout the experiment. The basic diet without herbs and not attacked (challenge) was used as non-challenged control (NC), and the base diet without herbs was infected with positive control (CC). Non-infected group with herbal medicine group (MNC), infected group with herbal medicine group (MCC). The study used a completely randomized design with a total of 4 treatments and 5 replicate cages with 10 chickens per cage (4^*^5^*^10). On day 28, all chickens except the NC and MNC groups were attacked with 2.5^*^10^4^ *E.tenella* Houghton (H) strains attack doses, which did not result in significant mortality but caused reduced growth performance and disruption of the intestinal epithelium for coccidial infection. Sporulated oocysts for chicken infection were washed 3 times with NaCl solution to remove potassium dichromate (K_2_Cr_2_O_7_) and then washed well with deionized water. The oocyst concentration was then adjusted to the desired concentration. 0.2 mL of saline solution was administered orally to the NC and MNC groups to produce the same administration pressure. Feed ingredients and the amount of drugs used (see Supplementary Material 1).

### Construction of a Database of the Target Sites of MGQD

“Magnolol, Puerarin, Baicalein, Berberine, Glycyyhizic acid” were searched in the TCM Systematic Pharmacology Database and Analysis Platform (TCMSP) (17), Drugbank (18) database to obtain currently known and reported target sites. The molecular structures provided by TCMSP are used in the PharmMapper server (19) and the Search Tool for Chemical Interactions (STITCH) (20) to obtain potential targets, summarize all drug targets to remove duplicate values, and build a complete drug target database. The Swiss Target Prediction database (21) predicts targets from a combination of 2D and 3D structural similarities of known small molecule substances. The way PharmMapper identifies potential drug targets is mainly by mapping the reverse pharmacophore.

### Construction of a Database of Differential Cecum Genes for *Eimeria tenella* Infection in Chickens

Using the SRA sub-database in the NCBI database (22), the cecum tissue transcriptome of chickens infected with Eimeria tenella was searched for and GSM5087811, GSM5087812, GSM5087813, GSM5087814, GSM5087815, GSM5087816, and analyze the differential genes (the above data are the published data of this laboratory). The differential genes were collated to construct a database of differential genes in the appendix of chickens infected with *Eimeria tenella*.

### Cross-Tabulation Analysis of MGQD and Differential Gene-Related Targets in the Cecum of Chickens Infected With *Eimeria tenella*

MGQD target protein database and chicken infection with *Eimeria tenella* cecum differential gene database were screened for intersecting genes using Venn diagram to identify potential target proteins for drug treatment of chicken coccidiosis. In order to more accurately express the potential target proteins for drug treatment of chicken coccidiosis, two datasets of MGQD and *Eimeria tenella*-infected chicken cecum differential genes were imported into Cytoscape, and the “Merge” tool was used to construct a “drug-disease” target network and to obtain cross-targets that could be used as candidate targets for further research. The complex relationships between MGQD histories, chicken coccidioidomycosis DEGs and target genes were visualized using Cytoscape 3.7.1 (22).

### Drug Target Protein-Chicken Coccidioidomycosis Differential Protein Interaction (PPI) Network Construction

The screened potential targets were imported into the STRING database (23), and the interaction relationships between the potential targets were analyzed to establish a drug target protein-chicken coccidioidomycosis differential protein interaction (PPI) network map. The NetworkAnalyzer function module in Cytoscape_v3.7.1 analyzes the topological parameters of the network, calculates the degree, betweenness, and closeness of the network, plots the size of the nodes and the thickness of the lines in the network based on the degree values, and determines the importance of each target point in the network. The analysis of the network can further elucidate the key drugs and targets. STRING is a database of knowing and predicted protein-protein interactions. Cytoscape is an open source software platform developed for biological research, mainly consisting of network annotation, gene expression profiling, and other state data integration. The network facilitates scientific explanations of the complex relationships between compounds, genes, pathways, and diseases.

### Analysis of Functional Processes and Molecular Pathways

To further obtain information on the biological functions and related biological pathways of each intersection target, all intersection targets were imported into Metascape and annotated to analyze the biological functions as well as the KEGG pathway, etc. Metascape database is a tool for gene function annotation analysis, which mainly contains functional modules such as KEGG pathway, GO biological process, reactive gene set, classical pathway, CORUM, TRRUST, DisGeNET, PaGenBase, transcription factor target, COVID, etc (24). In addition, the systematic data had satisfactory timeliness. During the analysis, words with *p* < 0.01, minimum count of 3, and enrichment factor >1.5 were collected and then grouped according to the similarity of their members (last update: 2021-12-10), and the roles and pathways of their intersection targets were summarized by software analysis.

### Screening for Hubba Gene

Evaluation of intersecting genes was carried out through the Hubba gene module in Cytoscape_v3.7.1 software to facilitate further data analysis. For the network topology analysis, the average distance from a node to other nodes measured by “maximum neighborhood component” (MNC) was applied. The level of the parameter shows the topological importance of the nodes in the network.

### Molecular Dockings

#### Preparation of the Molecular Structure of MGQD

The molecular structures of the “Magnolol, Puerarin, Baicalein, Berberine, Glycyyhizic acid” of MGQD were downloaded from the TCMSP database. The structures were converted to PDB format using Autodock4 software and selected as ligand in molecular docking (25).

#### Target Protein Preparation for MGQD

The top three common targets of MGQD and CC differential genes were selected according to the score ranking under the MNC algorithm in method 2.7, and the 3D structure of the target protein was downloaded from the PDB database, and the species selected was “Gallus gallus.” PDB format to.pdbqt format using Autodock. Hydrogen atoms are added, heteromolecules and water molecules are removed, and charge and bond order are assigned to the protein structure.

#### Molecular Docking With Autodock

Molecular ligands that have been processed with protein receptors were introduced into Autodock4 to demonstrate the reliability of the predicted potential targets, with the grid box size of each protein receptor determined according to the protein receptor size (Supplementary Material 2). The final assessment of ligand-protein binding is performed based on a sliding scale (Kcal/mol), selecting the ligand with the lowest Gibbs free energy to visualize ligand-protein docking in Pymol (26). Molecular docking 2D structures were demonstrated using Ligplus software (27). The degree of receptor-ligand binding can be judged by the energy level in the molecular docking results. In general, the lower the energy, the higher the likelihood of receptor-ligand interaction when the binding conformation of the compound to the receptor is stable. The stronger the predicted binding strength between the drug and the key target. Assessing component-target associations through molecular docking studies reduce network complexity and improve accuracy.

### Real-Time Quantitative Polymerase Chain Reaction

RT-QPCR was used to validate the expression levels of the top three MGQD and CC differential gene common targets scored under the MNC algorithm in method 2.7.RNA was extracted from cecum tissue using Spin Column Animal Total RNA Purification Kit (Sangon Iotech, China) according to the manufacturer's instructions, and the isolated RNA was synthesized using StarScript II First-strand cDNA Synthesis Mix With gDNA Remover StarScriptIIcDNA (GenStar, China) was used to reverse transcribe the isolated RNA samples to obtain cDNA. Then, cDNA and primers were added to the 2XRealStar Green Fast Mixtur (GenStar, China) system according to the manufacturer's protocol and real-time RT-QPCR was performed by the Light Cycler 480 Real-Time System (Roche, CA, USA). three cecum gene-related genes were normalized with internal reference genes. The mRNA levels of the three cecum gene-related genes were normalized with internal reference genes, the gene primer sequences for RT-QPCR (Supplementary Material 3), all RT-QPCRs were repeated three times, and the expression levels of candidate genes were analyzed by the 2-^Δ*ΔCT*^ method.

### Statistical Analysis

Statistically significant differences between the means of each experimental group were analyzed by using one-way ANOVA (one-way analysis of variance) followed by multiple comparisons using a *post-hoc* test of S-N-K with the statistical software IBM^®^ SPSS^®^ Statistics version 26. *P* < 0.05 were considered statistically significant. Graphs were plotted using Graph-Pad Prism-8.

## Result

### Clinical Indicators

**Figure 1:**
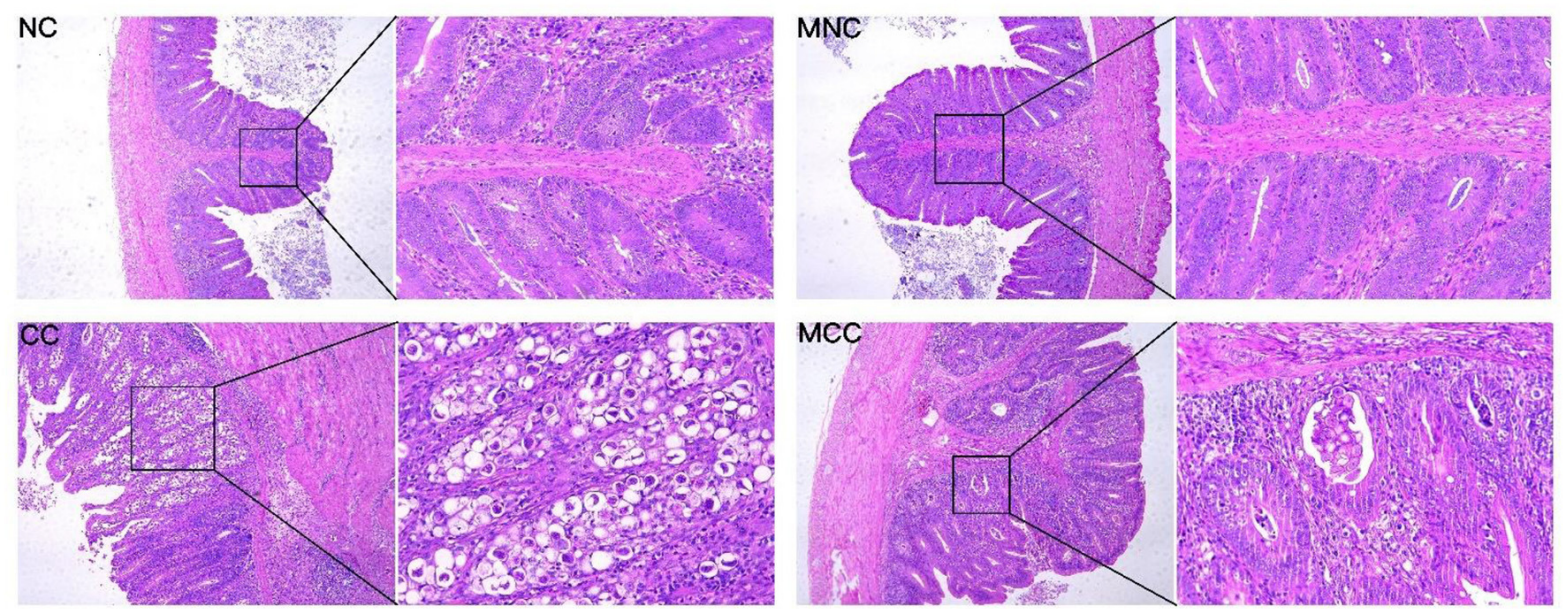
Sections of cecum lesions of chickens infected with *E. tenella* in different groups on day 7. Non-challenged control (NC), and the base diet without herbs was infected with positive control (CC). Non-infected group with herbal medicine group (MNC), infected group with herbal medicine group (MCC). The left side is the microscope 10X magnification display figure, the right side is the microscope 40X magnification display figure.

As observed in the infection-treated and untreated groups, the MGQD-treated group had lower scores for bloody diarrhea (5–7 dpi), oocyst shedding in the feces (132–192 hpi), and cecum lesions than the untreated CC group. no bloody diarrhea, oocyst shedding in the feces, or cecum lesions were found in either NC or MNC (Table 1). Figure 1 shows the microscopic examination of the cecum tissues. The base diet without herbs and not attacked (challenge) as non-challenged control (NC) and the herb-containing group without infection (MNC) were in normal condition, with a clear morphological structure of the cecum with a normal “finger-like bulge” and the merozoite of *Eimeria tenella* was not seen in the epithelial cells. The merozoite of *Eimeria tenella* was not seen in the epithelial cells of the epithelium and intestinal glands of the cecum of the positive control group (CC) infected on the basal diet without herbs, and atrophied villi were also observed in the glandular tissue disintegration and tissue autopsy. The herb-containing group of infected group (MCC) had fewer cecum lesions and only a small amount of the merozoite of *Eimeria tenella* was found.

**Table 1:** Effects of MGQD on bloody diarrhea scores, oocyst shedding, and lesion scores.

**Parameters**	**Groups**				
	**NC**	**MNC**	**CC**	**MCC**	**F**
**Lesion scores (Grade)**					
Day 7 post-infection	0.00 ± 0.00^b^	0.00 ± 0.00^b^	2.60 ± 0.89^a^	1.80 ± 0.84^a^	22.93
**Bloody diarrhea scores**					
5dpi	0.00 ± 0.00^b^	0.00 ± 0.00^b^	2.60 ± 0.89^a^	2.20 ± 0.45^a^	38.93
5.5dpi	0.00 ± 0.00^c^	0.00 ± 0.00^c^	3.60 ± 0.55^a^	2.40 ± 0.55^b^	108.00
6dpi	0.00 ± 0.00^c^	0.00 ± 0.00^c^	3.60 ± 0.55^a^	2.20 ± 0.45^b^	125.20
6.5dpi	0.00 ± 0.00^c^	0.00 ± 0.00^c^	3.00 ± 0.00^a^	1.80 ± 0.45^b^	216.00
7dpi	0.00 ± 0.00^c^	0.00 ± 0.00^c^	2.40 ± 0.55^a^	1.20 ± 0.45^b^	52.80
**Oocyst counting (OPG/g** **×10**^**6**^**)**					
132hpi	0.00 ± 0.00^c^	0.00 ± 0.00^c^	5.02 ± 0.07^a^	3.37 ± 0.15^b^	2679.36
144hpi	0.00 ± 0.00^c^	0.00 ± 0.00^c^	119.67 ± 2.52^a^	105.00 ± 3.00^b^	3319.91
156hpi	0.00 ± 0.00^c^	0.00 ± 0.00^c^	728.33 ± 4.16^a^	283.67 ± 2.08^b^	65520.04
168hpi	0.00 ± 0.00^c^	0.00 ± 0.00^c^	199.33 ± 2.08^a^	121.80 ± 0.72^b^	23725.85
192hpi	0.00 ± 0.00^c^	0.00 ± 0.00^c^	148.67 ± 1.53^a^	83.00 ± 1.00^b^	18688.10

*Superscript letters (^a−c^) show significant difference (p < 0.05). Results are presented as mean ± standard error of mean (n = 5). dpi, days post infection; hpi, hours post infection*.

### Search for Intersection Targets and Construction of PPI Network

**Figure 2:**
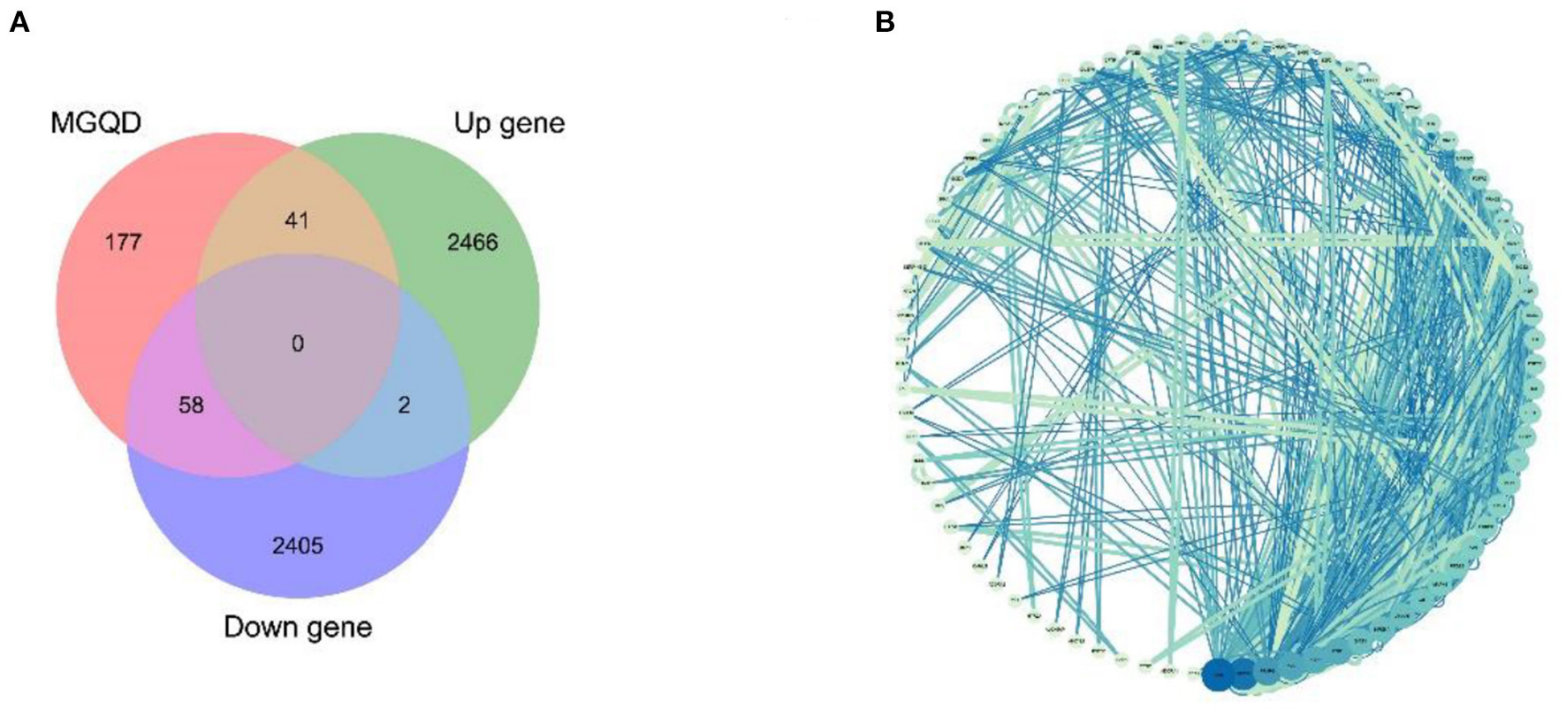
Drug target protein-chicken coccidioidomycosis differential protein interaction (PPI) network construction. **(A)** Number of potential targets of MGQD against coccidiosis in chickens. MGQD, database of the target sites of MGQD; Up gene, Up-regulated genes in transcriptome analysis of cecum tissue of chickens infected with *Eimeria tenella*; Down gene, Down-regulated genes in transcriptome analysis of cecum tissue of chickens infected with *Eimeria tenella*. **(B)** Potential target interactions of MGQD against chicken coccidiosis. The circle represents the intersection of MGQD and CC target proteins, the larger the diameter of the circle the darker the color, representing the greater the degree value. The line represents the interaction relationship between target proteins, the thicker the line the darker the color, indicating the stronger the interaction between target proteins.

Potential target proteins were collected by searching the database for each of the five drug components. In particular, 276 drug target proteins were collected by removing duplicates and replacing the gene name species with “Gallus gallus” using Uniprot to form the MGQD self-constructed database (Supplementary Material 4). After analyzing the DEGs data, a total of 2,464 up-regulated genes 2,509 down-regulated genes were obtained after comparison between the normal group and the challengers (Supplementary Material 5). The differential genes obtained from the analysis of RNA-seq were combined with two self-constructed target databases of MGQD drug targets, and the key targets of MGQD and chicken coccidiosis-related genes were identified by generating Venn diagrams. Ultimately, 99 genes were identified as targets of active ingredients and chicken coccidiosis-associated genes, the results are shown in Figure 2A. The STRING database was used to explore the interactions between potential key targets of MGQD for the treatment of chicken coccidiosis (Supplementary Material 6), and then imported into Cytoscape (version 3.7.1) to construct a visual PPI network containing 99 nodes and 165 edges for MGQD action on chicken coccidiosis, and the results are shown in Figure 2B. The targets are represented by circular nodes. The larger the node, the higher the degree, the brighter the color of the node, and the greater the mediator centrality. The larger and brighter the color of the node, the more important the target is in the network of “MGQD- Chicken coccidiosis.” The line thickness and color depth of the nodes indicate the magnitude of the edge mediator values. And the brighter the color of the connecting line between nodes and the thicker the line, the closer the interaction between the targets is indicated. The degree increase from light blue to dark blue, with larger nodes indicating higher degree and thicker edges indicating stronger interactions.

### Enrichment Analysis

**Figure 3:**
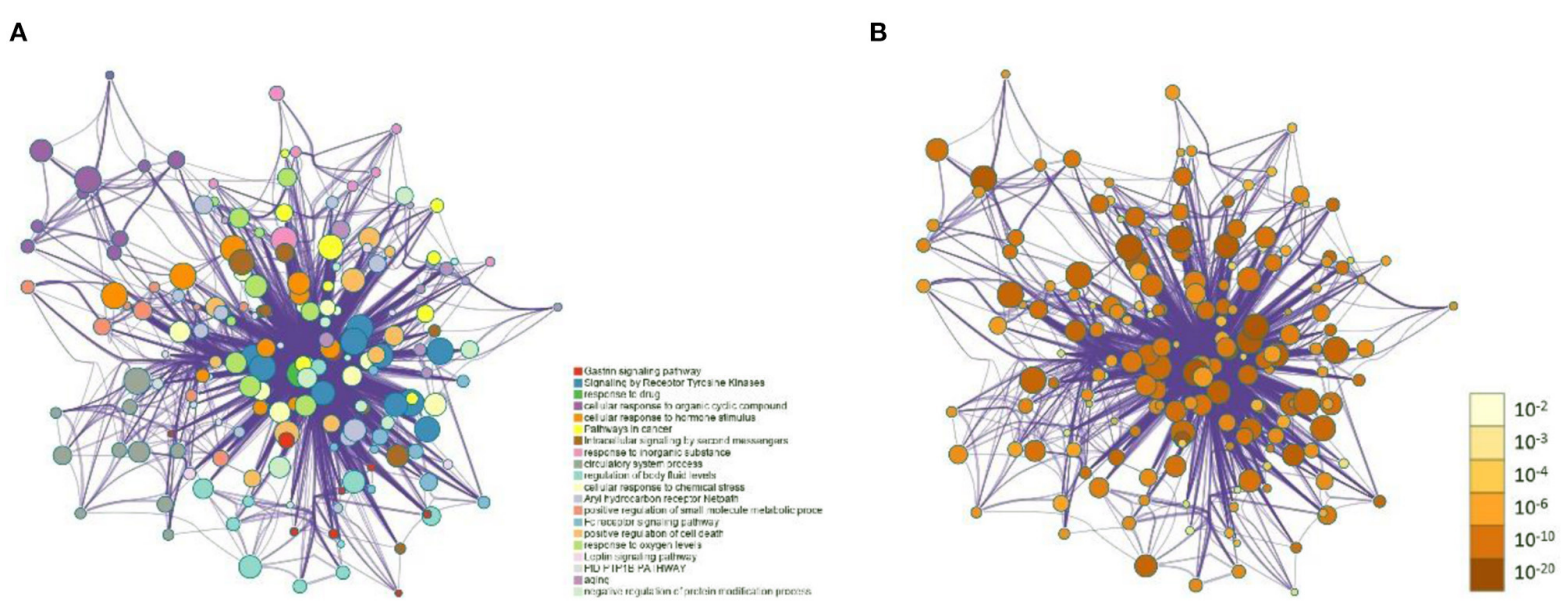
Network of enriched terms: **(A)** Circles represent enrichment to cluster IDs, colored by cluster ID, where nodes that share the same cluster ID are typically close to each other. **(B)** Network graph constructed according to the degree of enrichment, the darker the color, the more genes are enriched into the pathway.

The 99 potential key targets of MGQD against chicken coccidiosis were introduced into the Metascape platform, and the species was set as “Gallus gallus.” GO functional enrichment analysis and KEGG analysis were performed on the potential key targets. The top ranked GO annotation results and pathways were screened according to *p* (*p* < 0.01) values. The results are displayed by selecting the term content with the expected *p*-value from the 20 clusters. Supplementary Material 7 shows the richness of the relevant objective functions (top 20 ranking). To further determine the relationship between enriched terms, Kappa scores were calculated as a measure of similarity between words, and an enriched term similarity network was constructed, as shown in Figure 3. Cytoscape 3.7.1 was used to visualize the enrichment, where each node represents a term and is first colored according to its cluster ID (Figure 3A) and then its *p*-value (significance) (Figure 3B). The nodes form a network by the similarity (Kappa > 0.3) between the terms connected, and each node represents a rich term. It can be seen that terms belonging to the same cluster are more closely connected to each other. Indicates the degree of enrichment (*p*-value), and it can be seen that the higher the number of genes included, the more significant the *p*-value. For clarity, only one term label is shown for each cluster. After calculation, the network contains a total of 184 nodes and 2,559 edges. In this network, the degree is reflected by the size of the nodes. The larger the node, the higher the degree value and the shorter the distance between channels with similar functions.

**Figure 4:**
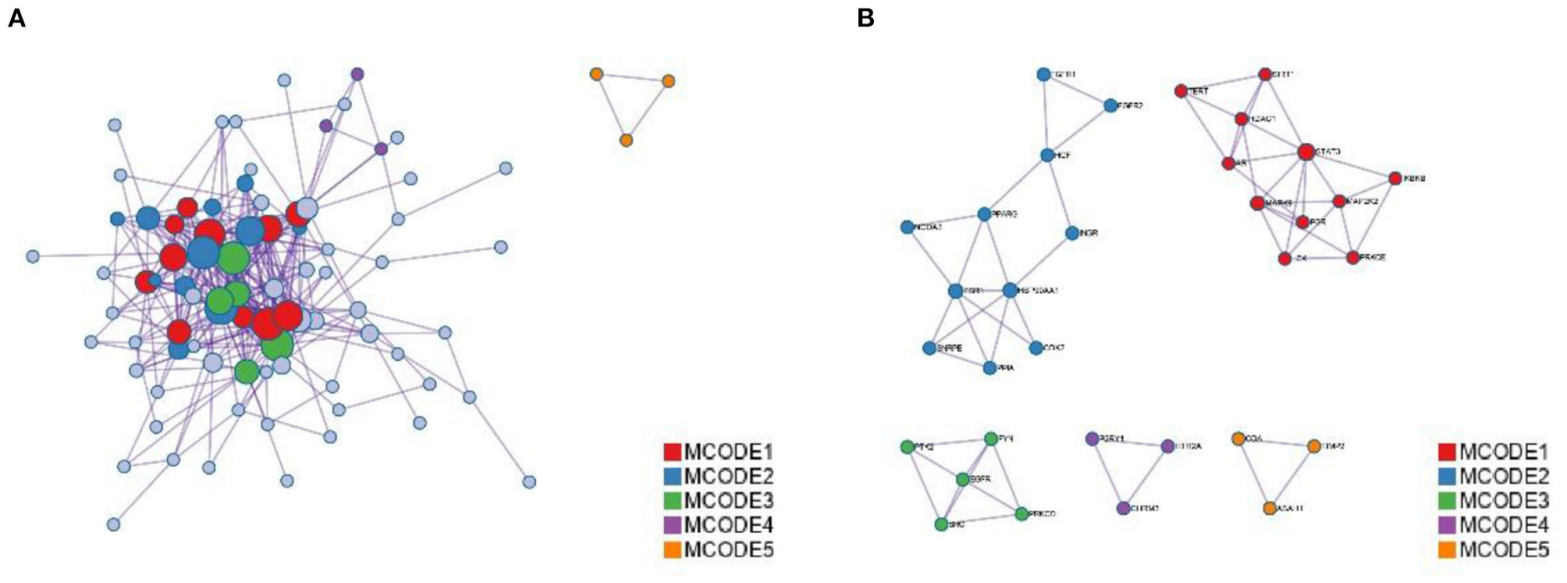
Protein-protein interaction enrichment analysis. **(A)** Protein-protein interaction networks identified in the gene list. Circles represent target proteins, the same color is an MCODE, forming a subset of interacting proteins. **(B)** Identified in the list of genes MCODE components.

The GO function enrichment analysis mainly involved three components: cellular composition, molecular function and biological processes, and by setting the filters to adjusted *p* < 0.05 and *q* < 0.05, we obtained 1,711 significantly enriched GO functions. The top 10 performances in Figure 4A were selected, respectively, for biological processes mainly involved in the regulation of cell proliferation and cellular processes, regulation of stimulus responses, and signaling, such as Regulation of cellular process (GO:0050794), Response to organic substance (GO:0010033) (GO:0050794), Response to organic substance (GO:0010033), Biological regulation (GO:0065007), Biological regulation (GO:0050789), and Biological regulation (GO:0007165). For cellular components, the target is enriched with Cellular anatomical entity, Cytoplasm, Intracellular, Cytosol, Organelle, Membrane-bounded organelle, Intracellular membrane-bounded organelle, Nucleus, Intracellular organelle, and Membrane etc. In terms of molecular function, MGQD treatment of chicken coccidiosis mainly involves the regulation of binding between ions, protein kinase activity, binding of compounds, Ion binding (GO:0043167), Protein kinase activity (GO:0004672), Protein tyrosine kinase activity (GO:0004713), Binding (GO:0005488), Organic cyclic compound binding (GO:0097159), Heterocyclic compound binding (GO:1901363), Catalytic activity (GO:0003824), Nucleotide binding (GO:0000166), Catalytic activity, acting on a protein (GO:0140096), and ATP binding (GO:0005524). The above biological processes or molecular functions can infer the pathogenicity of coccidiosis in chickens. GO terminology suggests that these target genes play an important role in host defense and stress response. The top 20 pathways are shown in Figure 4B, where the vertical coordinate represents the KEGG pathway of the target gene and the horizontal coordinate represents the enrichment factor, indicating the ratio of the number of target genes belonging to the pathway to the number of all annotated genes located in the pathway. A higher value of this ratio corresponds to a higher level of enrichment. In addition, the size of the dots indicates the number of target genes in the pathway. Gradually changing colors, from dark to light, represent the highest to lowest *p*-values, respectively.

**Figure 5:**
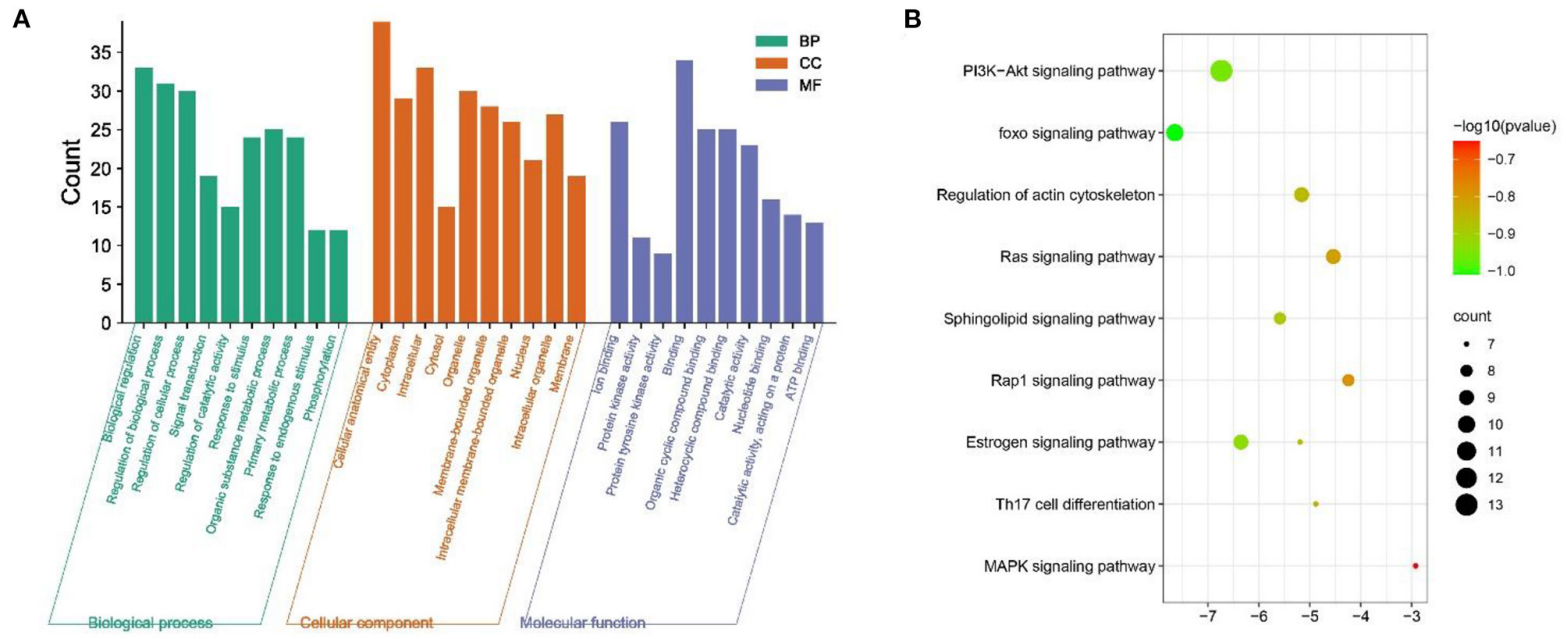
Analysis of functional processes and molecular pathways. **(A)** Top 10 Go features according to *P*-value. The bars represent the GO functions enriched to the intersection target proteins, the horizontal coordinate is sorted according to the *P*-value, and the vertical coordinate indicates the number of intersection targets present in the current GO function. **(B)** Top 10 KEGG pathways according to *P*-value. The circle represents the KEGG description on the left, the shade of color indicates the *P*-value, and the size indicates the number of intersecting targets present in the current KEGG pathway.

The annotation through the Metascape platform revealed that there are five different module substructures identified in the interaction network, formed by abstracting modules from the fully connected interaction network. The enrichment results show the key targets of MGQD acting on the protein-protein interaction network of chicken coccidiosis and the MCODE component of the identified gene list, indicating that the relevant targets may be more prominent, the results are shown in Figure 5A. Five of these clustering modules with *p* < 0.05 suggest that the target proteins in them are more closely related and may work together to perform a common biological process. As shown in Figure 5B, cluster 1 was associated with PID IL2 1PATHWAY and MicroRNAs in cancer, cluster 2 was associated with PIP3 activates AKT PI5P, PP2A, and IER3 Regulate PI3K/AKT Signaling, and Negative regulation of the PI3K/AKT network, cluster 3 was associated with Gastrin signaling pathway, Fc-gamma receptor signaling pathway involved in phagocytosis, immune response-regulating cell surface receptor signaling pathway involved in phagocytosis, cluster 4 was associated with blood vessel diameter maintenance, regulation of tube diameter, and regulation of tube size, cluster 5 was associated with Neutrophil degranulation.

**Figure 6:**
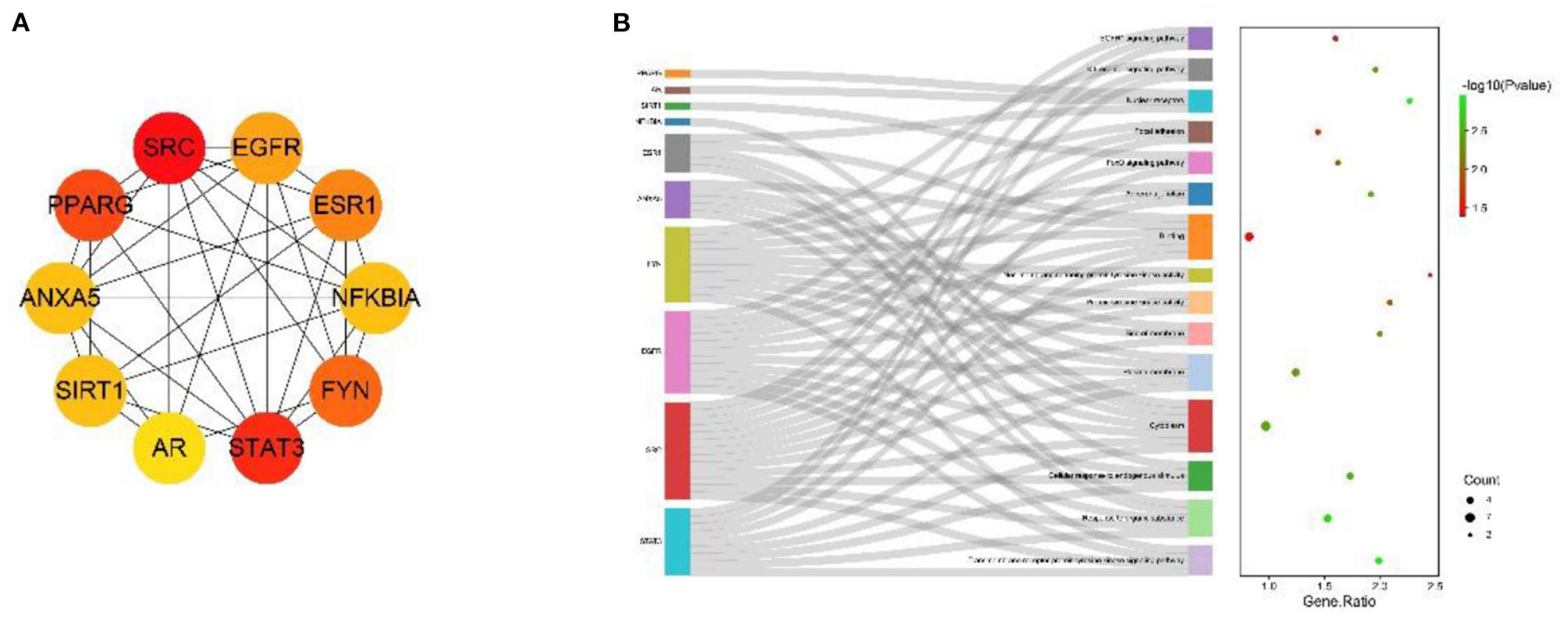
The screened Hubba genes and the results of their enrichment analysis. **(A)** The 10 central genes identified from the PPI network. Circles represent target proteins, with darker colors indicating higher MNC scores and greater importance in intersecting target proteins. **(B)** The first 3 GO terms and KEGG pathways for the 10 Hubba genes. The sankey diagram represents the correspondence between the top 10 genes according to the MNC score (left) and the enriched GO function and KEGG pathway (right). The circles in the bubble diagram represent functional descriptions, the shade of color indicates the size of the *P*-value, and the size of the diameter indicates the number of intersecting targets present in the current GO function or KEGG pathway.

The MNC algorithm was used to filter the hub genes, and the top 10 core nodes were identified and ranked in the “Cyto Hubba” plug-in, as shown in all the score files in Supplementary Material 8. The results are specifically depicted in Figure 6A, and showed that SRC, STAT3, PPARG, FYN, ESR1, SIRT1, EGFR, SOD3, LCK, and PRKCE are the key targets under the MNC algorithm. In addition, GO and KEGG analyses were performed again on 10 central genes to verify their biological functions. From the Sankey diagram (Figure 6B), it was found that the key targets were mainly focused on EGFR1 signaling pathway, Kit receptor signaling pathway and FoxO signaling pathway.

### Molecular Docking and Analysis

The computational molecular docking binding can be studied to assess the affinity of the component to the target target, reducing network complexity, and improving accuracy. The binding energy reflects the possibility of receptor-ligand binding. The lower the binding energy, the higher the affinity of the receptor to the ligand and the more stable the conformation. The results of the docking binding energy (Supplementary Material 9) reveal that these drugs can bind well to the active site of the protein target. Among them, SRC had the lowest docking binding energy with Berberine (−6.35 kcal/mol), STAT3 had the highest binding energy with Glycyyhizic acid (−3.51 kcal/mol), and the average binding energy was −4.80 kcal /mol, reflecting the fact that all three candidate compounds had good or even strong binding activity with each candidate protein target, respectively. The average binding energy was −4.80 kcal/mol, reflecting the good and even strong binding activity in the molecular docking between all three candidate compounds and each candidate protein target, respectively.

**Figure 7:**
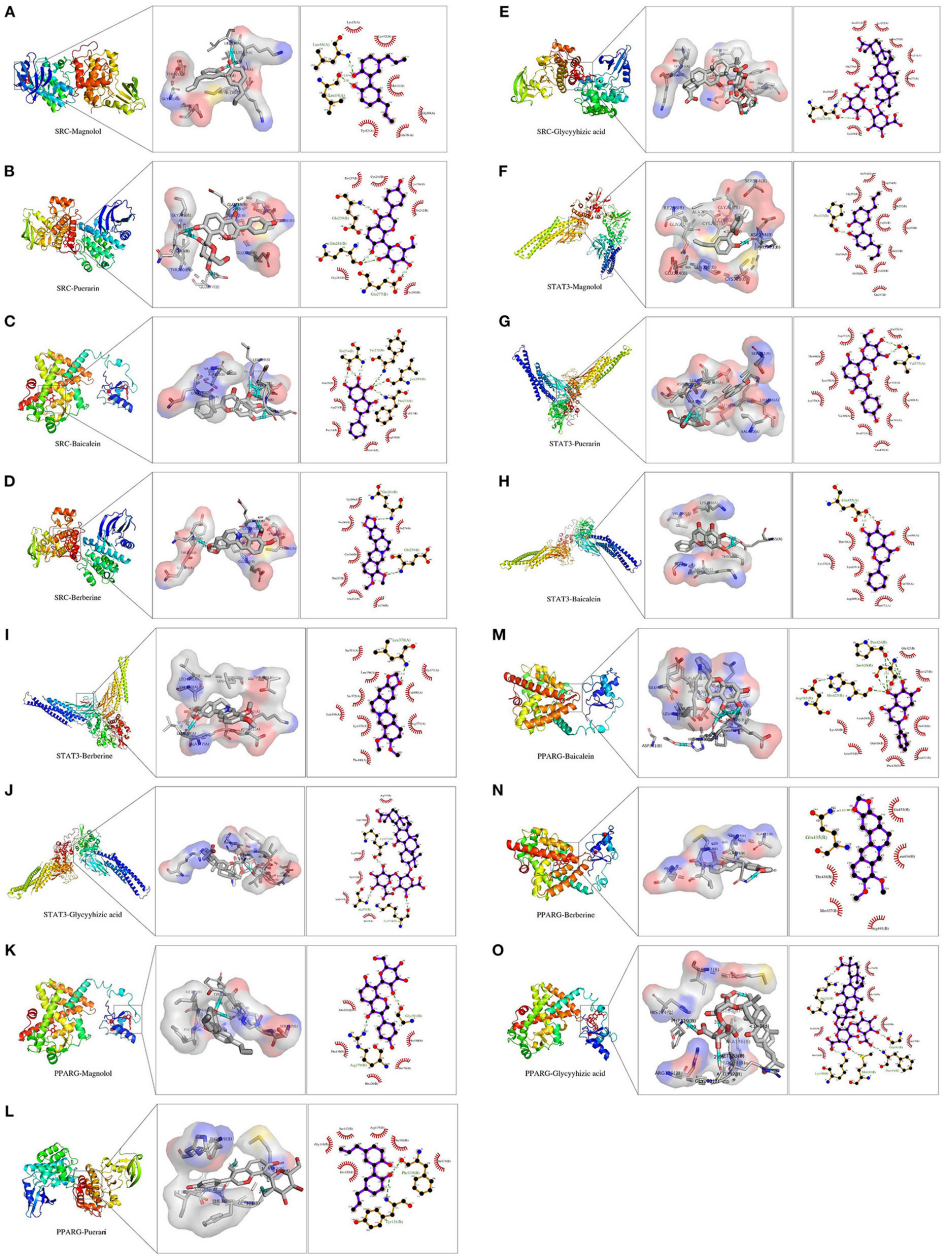
**(A–O)** Demonstration of molecular docking of drugs and Hubba genes. **(A)** Demonstration of molecular docking of SRC and Magnolol. **(B)** Demonstration of molecular docking of SRC and Puerarin. **(C)** Demonstration of molecular docking of SRC and Baicalein. **(D)** Demonstration of molecular docking of SRC and Berberine. **(E)** Demonstration of molecular docking of SRC and Glycyrrhizic acid. **(F)** Demonstration of molecular docking of STAT3 and Magnolol.(G) Demonstration of molecular docking of STAT3 and Puerarin. **(H)** Demonstration of molecular docking of STAT3 and Baicalein. **(I)** Demonstration of molecular docking of STAT3 and Berberine. **(J)** Demonstration of molecular docking of STAT3 and Glycyrrhizic acid. **(K)** Demonstration of molecular docking of PPARG and Magnolol. **(L)** Demonstration of molecular docking of PPARG and Puerarin. **(M)** Demonstration of molecular docking of PPARG and Baicalein. **(N)** Demonstration of molecular docking of PPARG and Berberine. **(O)** Demonstration of molecular docking of PPARG and Glycyrrhizic acid. The left side is the complete 3D stereo image of molecular docking, the middle is the 3D detail display image of molecular docking, and the right side is the 2D detail display image of molecular docking.

The results showed that SRC interacted with Magnolol, Puerarin, Baicalein, Berberine, and Glycyyhizic acid, as shown in Figures 7A–E. As shown in Figure 7A, the structure of Magnolol can interact with Leu16(A) and Leu14(A) through three hydrogen bonds, respectively, and with Ser292 through one hydrogen bond in the SRC. As shown in Figure 7B, the structure of Puerarin can form 1 hydrogen bond, 2 hydrogen bonds and 1 hydrogen bond with Glu239 (B), Gln281 (B), and Glu277 (B), respectively, in the SRC. As shown in Figure 7C, Baicalein can interact with Thr274 (B), 1 hydrogen bond with Thr272 (B), 1 hydrogen bond with Leu269 (B) and 1 hydrogen bond with Phe273 (B) via 3 hydrogen bonds, respectively. As shown in Figure 7D, Berberine can interact with Glu239 (B) and Gln281 (B) in SRC through 1 hydrogen bond, respectively. As shown in Figure 7E, Glycyyhizic acid can interact with Gln281 (B) in SRC through 2 hydrogen bonds.

The results showed that PPARG interacted with Magnolol, Puerarin, Baicalein, and Berberine with Glycyyhizic acid, as shown in Figures 7F–J. As shown in Figure 7F, the structure of Magnolol can interact with Gly181 (B) and Arg179 (B) in SRC through 1 hydrogen bond, respectively. As shown in Figure 7G, the structure of Puerarin can interact with Phe119 (B) and Tyr121 (B) through a hydrogen bond, respectively. As shown in Figure 7H, 2 hydrogen bonds can bind Baicalein to Pro424(B) and Ser426(B) in PPARG, respectively, and 1 hydrogen bond interacts with His423(B). As shown in Figure 7I, the structure of Berberine can interact with Gln435 via one hydrogen bond to PPARG. Figure 7J, the structure of Glycyyhizic acid interacts with PPARG via one hydrogen bond to Arg182 (B), Lys190 (B), Met183 (B), Phe119 (B), and Gly181 (B).

The results showed that STAT3 interacted with Magnolol, Puerarin, Baicalein, and Berberine with Glycyyhizic acid, as shown in Figures 7K–O. As shown in Figure 7K, the structure of Magnolol can interact with Pro333 (B) via a 1 hydrogen bond. As shown in Figure 7L, the structure of Puerarin can interact with Val375 (A) via 2 hydrogen bonds. As shown in Figure 7M, 2 hydrogen bonds can interact Baicalein with Glu455(A) in STAT3, respectively. As shown in Figure 7N, the structure of Berberine can interact with Leu378(A) in STAT3 through 1 hydrogen bond. Figure 7O, the structure of Glycyyhizic acid interacts with STAT3 by a hydrogen bond to Lys573 (B), Ala578 (B), and Lys574 (B), respectively. Thus, there is an interaction between these compounds and protein targets with hydrogen bonds.

### Analysis of Hubba Gene Expression Levels

**Figure 8:**
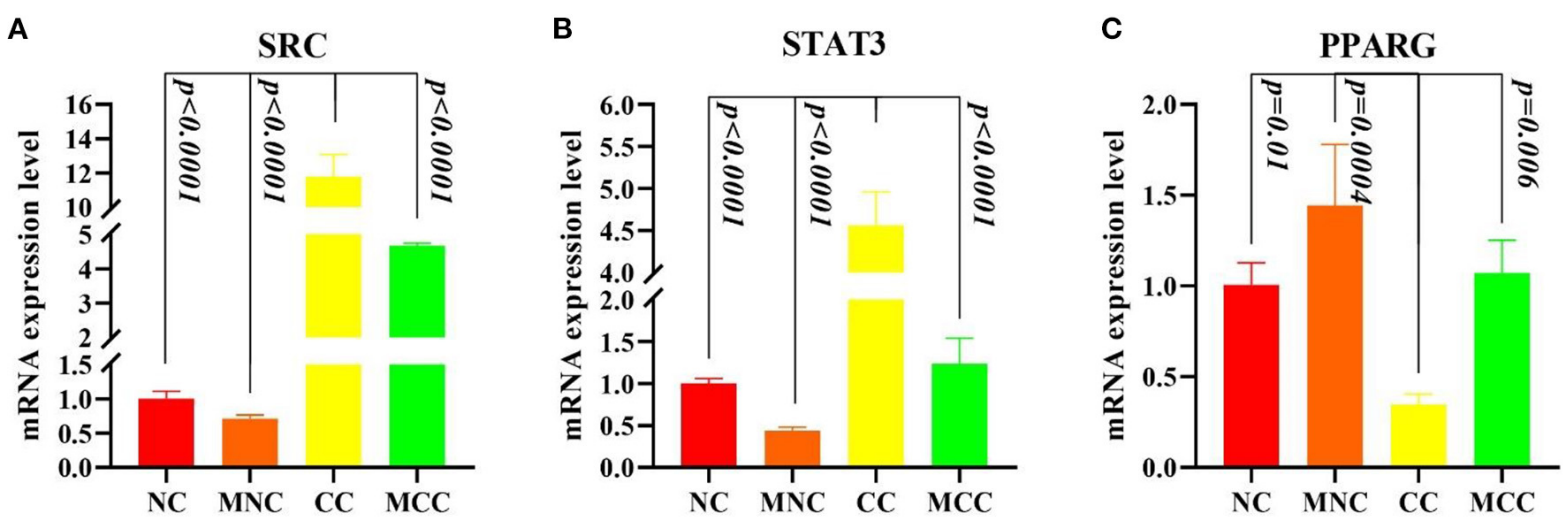
**(A–C)** Expression levels of Hubba gene mRNA. **(A)** Expression levels of SRC mRNA expression level. **(B)** Expression levels of STAT3 mRNA expression level. **(C)** Expression levels of PPARG mRNA expression level. The difference was significant if *p* < 0.05 compared to the control group.

Figure 8 shows that infection with *Eimeria tenella* elevated the expression of SRC and STAT3 and decreased the expression of PPARG in CC vs. NC compared to normal control (*p* < 0.0001, *p* < 0.0001, and *p* = 0.01, respectively), consistent with the transcriptome results, and MGQD pretreatment results vs. NC group indicated no significant difference in the expression of SRC, STAT3, and PPARG key targets. The expression of SRC and STAT3 was significantly reduced and the expression of PPARG was increased in the group receiving the treatment with the addition of MGQD compared with the model group (*p* < 0.0001, *p* < 0.0001, and *p* = 0.006, respectively).

## Discussion

It is important to prevent chicken coccidiosis from affecting the growth performance of chickens. It is one of the most common parasitic diseases in modern intensive poultry farming and is caused by intracellular parasitic protozoa of the genus *Eimeria* (5). However, there is a lack of drugs, and traditional treatment drugs tend to lead to the development of drug resistance, aggravate environmental pollution, and are not as effective (4). Gegen Qinlian Decoction, a traditional Chinese remedy, has a wide range of biological activities, and Magnolia officinalis has also been shown to have some anthelmintic effects. Studies by others and our group have previously shown that MGQD may improve disorders of lipid metabolism and has the potential to treat ulcerative colitis (28). The report showed that the combination of the principles of Chinese veterinary diagnosis and treatment of formulae for the control of coccidiosis in livestock and poultry obtained a better control effect and achieved good results in its treatment, and the use of Chinese herbal medicine to solve coccidiosis in livestock and poultry is not a good method (5). Most of the western drug products such as monensin, maduramicin and salinomycin, which are widely used in the market today, have developed resistance in poultry, and the resistance developed in poultry food has been shown to be hazardous to public health (29). The natural plant-based feed additives have the advantages of low cost, natural residue-free and no drug resistance compared to the above drugs (30). Therefore, herbal preparations have great potential in the control of coccidiosis in chickens. However, as far as the current studies are concerned, the research on the control of herbal medicines for chicken coccidiosis is still mainly confined to the comparison of efficacy with western medicines and field trials, and the specific drug effects (whether they are effective against inflammation) and mechanisms of action still need to be further studied.

Network pharmacology has been widely used in human diseases, but not in animal diseases been well-applied in animal diseases. Network pharmacology can provide an understanding of all or part of the principles of network theory and systems biology, making it a cutting-edge approach to drug discovery. This approach has been used to study the “compound-protein/gene-disease” pathway, capturing the complexity between biological systems, drugs, and diseases from a network perspective. Therefore, the development of network pharmacology that can predict multiple drug-target interactions may be key to the future successful development of drugs against complex diseases such as chicken coccidiosis. In our study, the corresponding targets in human diseases were replaced with genes that showed differences in transcriptomic expression, followed by subsequent analysis, and docking validation was added to network pharmacology as a complement to predictive drug targets.

In this study, we applied a systems pharmacology approach to predict and elucidate the potential molecular mechanisms of action of MGQD active substances on chicken coccidiosis. RNA-seq identified genes and pathways activated in response to chicken coccidiosis that are involved in the intervention of *Eimeria tenella* on the animal organism. The PC group had 2509/2465 DEGs compared to the NC group, respectively, meeting the criterion of *P* < 0.05. We performed a composite target and target pathway network analysis and identified 99 key genes in the effective active substance-target network of MGQD to construct a protein interaction network. GO enrichment analysis of 99 targets showed that the therapeutic effects of MGQD on chicken coccidiosis mainly involved the regulation of cell proliferation and cellular processes, the regulation of stimulatory responses and signaling, and in terms of molecular functions, MGQD treatment of chicken coccidiosis mainly involved the regulation of binding between ions, the activity of protein kinases, and the binding of compounds. Processes such as cell proliferation apoptosis, enzyme activation and compound binding play an important role in the pathophysiology of C. flexneri on host cells (31, 32). Yan Zhang et al. found that E. tenella infected chicken embryonic cecum epithelial cells could reduce the apoptosis rate at the early stage of development to protect the host cells, and as *Eimeria tenella* developed in the host cells, it led to an increase in the apoptosis rate which was detrimental to the intracellular growth and development of *E. tenella* (32). ZhiYong Xu et al. found that *E. tenella* inhibited host cell apoptosis through the TNF receptor-associated death domain -RIP1 pathway in early development and promoted host cell apoptosis through the TNF receptor-associated death domain -Caspase-8 pathway in mid- to late-development by using Gene silencing. death domain -Fas-associated death domain -caspase-8 apoptosis pathway to promote host cell apoptosis (33). Studies show that the kinase of rhoptry compartment (ROP) is a key virulence factor in Toxoplasma gondii by hijacking and regulating many cellular functions and pathways to infect cells (30). While in *E. tenella* infection or EtROP1 overexpression affects p53 interactions and phosphorylation, ultimately leading to host cell apoptosis and inhibition of G0/G1 cell cycle arrest (34). Dexing Ma realized that specific L peptides, competing with E. tenella rhotryneck protein 2 (EtRON2) to bind EtAMA1 located on the surface of ascospores, inhibited the parasite's invasion of cells (35). It was shown that EtSTP protein acts mainly in the developmental and reproductive stages of the parasite and that the ability of ascospores to invade cells was significantly reduced by anti-rEtSTP antibody treatment (36). Anti-EtMIC8 antibodies significantly increase the rate of adhesion to host cells and reduce the rate of invasion of ascospores into host cells (37). Our results predict that MGQD affects molecules such as Ion binding, Protein kinase activity, Protein tyrosine kinase activity, and Binding at least by regulating cellular components such as Cytoplasm, Intracellular, and Cytosol The effect of the product is to further regulate the biological processes such as Regulation of biological process, Regulation of cellular process, Signal transduction, Regulation of catalytic activity, etc., to achieve a control of chicken coccidiosis. The effect of the study was achieved.

In the results of KEGG enrichment analysis (38), it was observed that some signaling pathways (e.g., PI3K-Akt signaling pathway, Foxo signaling pathway, Estrogen signaling pathway, MAPK signaling pathway, Th17 cell differentiation) are closely associated with the development and metastasis of chicken coccidioidomycosis. There is evidence that activation of the PI3K/Akt signaling pathway inhibits apoptosis in *E. tenella* host cells early in infection by reducing the expression of bad content, limiting the opening of the membrane permeability transition pore, and decreasing Caspase-9 and Caspase-3 activity (39). The FoxO transcription factor family regulates the expression of genes in cellular physiological events, including apoptosis (40), glucose metabolism (41), longevity (42–44), maintenance of tissue homeostasis in the animal organism and coordination of responses to environmental changes (45). There is also growing evidence that *Eimeria tenella* infected animals develop disease that is primarily mediated by inflammatory pathways. The expression profile of miRNAs in the intestinal tissues of Hy-line strains of white chickens infected with *Eimeria tenella* was studied by deep sequencing, and a total of 35 miRNAs were significantly differentially expressed, and pathways related to cell proliferation and apoptosis were identified, such as MAPK signaling pathway and PPAR signaling pathway (46). A hallmark of Th17 cells is the production of IL-17A (also known as IL-17), a pro-inflammatory cytokine. Lei Zhang found that chickens in the E. tenella-infected IL-17-neutralized group had increased growth performance and histopathology also showed reduced neutrophil recruitment and reduced parasite counts, thus concluding that IL-17 may mediate *E. tenella*-induced immunopathology during the infection (47).

Detection of densely connected regions in large protein-protein interaction networks that may represent molecular complexes. The method is based on vertex weighting of local neighborhood densities and outward traversal of locally dense seed proteins to isolate dense regions according to given parameters. The algorithm has the advantage over other graph clustering methods of having a directed mode that allows fine-tuning the clusters of interest without considering the rest of the network and allows checking the cluster interconnectivity associated with the protein network. Modules are a group of closely related proteins that act in concert to perform specific biological functions through protein-protein interactions that occur in time and space. In this study, the PPI network was divided into three clustering modules using the MCODE plug-in. Among them, module 1 contains almost all the key target genes and its score is the highest, so it is considered as the core clustering module. In addition, the module was significantly enriched with various genes closely related to inflammation, such as CDK2, PPA, ESR1, FGFR1, and FGFR2, theoretically suggesting that it may be related to inflammation-related processes.

In order to demonstrate the protein interactions more intuitively, the topological characteristics of the nodes in the PPI network were analyzed, and SRC, STAT3, PPARG, FYN, ESR1, EGFR, SIRT1, ANXA5, NFKBIA, and AR were screened based on MNC for MGQD treatment of chicken coccidiosis, and it was hypothesized that these 10 targets play key role in MGQD treatment of chicken coccidiosis. The first 3 core genes were SRC, STAT3, and PPARG. We compared the gene expression levels of SRC, STAT3, and PPARG and the statistical relationships between NC and CC. The results of differential expression analysis confirmed that SRC and STAT3 up-regulated PPARG were among the down-regulated genes in CC samples, with significant differences between samples (*p* < 0.05). Further molecular docking experiments in this study showed that Berberine has strong binding activity to SRC, STAT3, and PPARG, Magnolol bind strongly to STAT3 and PPARG, and Baicalein binds strongly to SRC and PPARG. Molecular docking results showed that Berberine, Magnolol and Baicalein have superior affinity for the target genes SRC, STAT3, and PPARG, and are active ingredients with potent anti-inflammatory and antioxidant effects. Berberine exerts its κB signaling pathway by blocking IL-6/STAT3/NF-local anti-inflammatory effects. STAT3 and PPARG are the targets associated with most of the active ingredients. The validity of the association was also verified by the results of molecular docking. Non-receptor protein tyrosine kinase (SRC), activated upon binding to many different types of cellular receptors, plays a key role in signaling pathways involved in the control of multiple biological activities and is also active at cell-cell contact adhesion sites and gap junctions, and also has a metastable network of binding substrate binding sites (48). As early as 1997 Williams J C had discovered the crystal structure of chicken Src, which is similar to that of human Hck and Src (49). According to Li H, SRC is a key host regulator controlling host-ILTV interactions, and Src prolongs host cell survival by increasing the threshold for virus-induced cell death, thereby identifying a positive feedback loop between Src and tyrosine kinase adherent spot kinase (FAK) (50). Wang Z showed by further genome-wide transcriptional profiling combined with functional validation that Src controls the cell-to-cell spread of ILTV in a regulatory cellular fatty acid metabolism-dependent manner, which determines the cytopathic effects of the virus (51). STAT3 belongs to the STAT family, and STAT3 is involved in a variety of signaling pathways and plays a signaling role. It is a marker transcription factor that binds to the interleukin 6 (IL-6) response element in the promoters of different acute phase protein genes. STAT3 may affect CAM angiogenesis and embryonic growth in the female chick embryo by mediating the VEGF/NO pathway (52). MDV virus infection activates the role of STAT3 in interrupting the ATR-Chk1 pathway during MDV replication (53). When Stat3ER is artificially activated, Jak1 inhibition is eliminated and dense colony formation is restored (54). Binding peroxisome proliferator (PPARG) controls the peroxisomal β-oxidation pathway of fatty acids. Therefore, Tunim S hypothesized that PPARG activity plays a key role in lipid accumulation through upregulation (55). Praud C's results based on innovative histological techniques of fluorescence intensity measurements suggest that the balance between TGFB1 and PPARG is essential for fibrosis or steatosis induction (55). Acts as a key regulator of intestinal homeostasis by inhibiting NF-κ-B-mediated pro-inflammatory responses. Plays a role in the regulation of cardiovascular circadian rhythms by regulating ARNTL/BMAL1 transcription in the vasculature [57]. In addition, MGQD showed good efficacy in animal experiments, and based on the results of network pharmacology three keys are considered to be the key targets of MGQD against chicken coccidiosis, and RTPCR relative quantification experiments were conducted on the key three target genes. Our data showed that CC elevated the expression of SRC and STAT3 and decreased the expression of PPARG in comparison with NC, consistent with the transcriptome results. MGQD pretreatment results compared with the NC group indicated no significant difference in the expression of SRC, STAT3, and PPARG key targets, further confirming MGQD control of *Eimeria tenella*-induced key targets in chicks. These results suggest that MGQD controls *Eimeria tenella*-induced coccidiosis in chickens mainly through fatty acid metabolism and oxidation, acting on inflammatory pathways to hinder intracellular parasitism of *Eimeria tenella*.

## Conclusion

This study indicates that the mechanism of action of MGQD in the treatment of chicken coccidiosis is related to its anti-inflammatory and antioxidant properties and inhibition of oncogene transcription. Meanwhile, action targets were related to SRC, STAT3, and PPARG. Therefore, a follow-up experimental analysis is worthwhile to confirm the reliability of the results.

## Data Availability Statement

The datasets presented in this study can be found in online repositories. The names of the repository/repositories and accession number(s) can be found in the article/Supplementary Material.

## Ethics Statement

The animal study was reviewed and approved by Experimental Animal Ethics Committee of Guangxi University.

## Author Contributions

XP and KW contributed to conception and design of the study. XP organized the database and wrote the first draft of the manuscript. KW performed the statistical analysis. YuW, YL, FL, YC, and YiW wrote sections of the manuscript. All authors contributed to manuscript revision, read, and approved the submitted version.

## Funding

The Key Research and Development Plan of Guangxi, China (AB19245037), Natural National Science Foundation of China (317607446), and the Major R&D Project of Nanning (20212138).

## Conflict of Interest

The authors declare that the research was conducted in the absence of any commercial or financial relationships that could be construed as a potential conflict of interest.

## Publisher's Note

All claims expressed in this article are solely those of the authors and do not necessarily represent those of their affiliated organizations, or those of the publisher, the editors and the reviewers. Any product that may be evaluated in this article, or claim that may be made by its manufacturer, is not guaranteed or endorsed by the publisher.
