# Supplementary material for: Exploration of the Mechanism of the Control of Coccidiosis in Chickens Based on Network Pharmacology and Molecular Docking With the Addition of Modified Gegen Qinlian Decoction
Source: Front Vet Sci. 2022 Mar 17;9:849518. doi: 10.3389/fvets.2022.849518 (PMC8968990; doi:10.3389/fvets.2022.849518)
Supplement: Supplementary file 1 [file Data_Sheet_1.Zip › Supplementary Table.docx]

| Items | NC/CC | MNC/MCC |
| --- | --- | --- |
| Corn | 59.1 | 59.1 |
| Soybean meal | 30.6 | 30.6 |
| Corn gluten meal | 3.8 | 3.8 |
| Vegetable oil | 1.7 | 1.7 |
| Limestone | 1.31 | 1.31 |
| Calcium phosphate | 1.77 | 1.77 |
| Sodium chloride | 0.42 | 0.42 |
| l-Lysine | 0.15 | 0.15 |
| DL-methionine | 0.15 | 0.15 |
| Premixa | 1 | 1 |
| Magnolol | 0 | 0.0260 |
| Berberine | 0 | 0.0094 |
| Glycyyhizic acid | 0 | 0.0063 |
| Baicalein | 0 | 0.0094 |
| Puerarin | 0 | 0.0094 |

**Table S1. Feed ingredients and the amount of drugs used**

**Table S2. Gridcenter**

|  | SRC | STAT3 | PPARG |
| --- | --- | --- | --- |
| X | -67.992 | -43.906 | 2.574 |
| Y | 36.0 | -43.903 | 41.042 |
| Z | -37.308 | 7.958 | 3.606 |

**Table S3: RT-qPCR primer sequence for validation of network pharmacology**

| Genes | Primer name | Primer Sequence (5_3_) |
| --- | --- | --- |
| *GAPDH* | GAPDH-F | TGATGCCCCCATGTTTGTGA |
|  | GAPDH-R | TGGCATGGACAGTGGTCATA |
| *SRC* | SRC-F | CACCACTTTCGTGGCTCTCT |
|  | SRC-R | CACCAGTCACCTTCCGTGTT |
| *STAT3* | STAT3-F | ACCTGTACCCAGACATCCCA |
|  | STAT3-R | ATACGGAGCAGCACTACCTG |
| *PPARG* | PPARG-F | TGTCGCATCCATAAGAAAAGC |
|  | PPARG-R | GTCCAAACCCGCTTACG |

**Table S7. Top 20 clusters with their representative enriched terms (one per cluster)**

| Category | Description | Count | % | Log10(P) | Log10(q) |
| --- | --- | --- | --- | --- | --- |
| WikiPathways | Gastrin signaling pathway | 14 | 14.14 | -17.40 | -13.24 |
| Reactome Gene Sets | Signaling by Receptor Tyrosine Kinases | 22 | 22.22 | -17.13 | -13.24 |
| GO Biological Processes | response to drug | 20 | 20.20 | -16.99 | -13.23 |
| GO Biological Processes | cellular response to organic cyclic compound | 22 | 22.22 | -16.07 | -12.51 |
| GO Biological Processes | cellular response to hormone stimulus | 22 | 22.22 | -16.03 | -12.51 |
| KEGG Pathway | Pathways in cancer | 19 | 19.19 | -15.81 | -12.35 |
| Reactome Gene Sets | Intracellular signaling by second messengers | 17 | 17.17 | -15.12 | -11.76 |
| GO Biological Processes | response to inorganic substance | 21 | 21.21 | -15.10 | -11.76 |
| GO Biological Processes | circulatory system process | 20 | 20.20 | -13.64 | -10.42 |
| GO Biological Processes | regulation of body fluid levels | 17 | 17.17 | -13.60 | -10.41 |
| GO Biological Processes | cellular response to chemical stress | 16 | 16.16 | -13.20 | -10.10 |
| WikiPathways | Aryl hydrocarbon receptor Netpath | 9 | 9.09 | -13.07 | -10.03 |
| GO Biological Processes | positive regulation of small molecule metabolic process | 12 | 12.12 | -12.93 | -9.95 |
| GO Biological Processes | Fc receptor signaling pathway | 9 | 9.09 | -12.90 | -9.94 |
| GO Biological Processes | positive regulation of cell death | 19 | 19.19 | -12.31 | -9.43 |
| GO Biological Processes | response to oxygen levels | 15 | 15.15 | -11.84 | -9.05 |
| WikiPathways | Leptin signaling pathway | 9 | 9.09 | -11.18 | -8.45 |
| Canonical Pathways | PID PTP1B PATHWAY | 8 | 8.08 | -11.01 | -8.31 |
| GO Biological Processes | aging | 14 | 14.14 | -10.81 | -8.13 |
| GO Biological Processes | negative regulation of protein modification process | 16 | 16.16 | -10.42 | -7.80 |

**Table S8. Top 10 in network string interactions ranked by MNC method**

| Rank | Name | Score |
| --- | --- | --- |
| 1 | SRC | 33 |
| 2 | STAT3 | 31 |
| 3 | PPARG | 24 |
| 4 | FYN | 23 |
| 5 | ESR1 | 22 |
| 6 | EGFR | 21 |
| 7 | SIRT1 | 19 |
| 7 | ANXA5 | 19 |
| 7 | NFKBIA | 19 |
| 10 | AR | 18 |

**Table S9. Binding Energy**

|  | Magnolol | Puerarin | Berberine | Baicalein | Glycyyhizic acid |
| --- | --- | --- | --- | --- | --- |
| SRC | 4.61 | 3.97 | 6.35 | 5.04 | 4.64 |
| STAT3 | 5.11 | 4.59 | 5.85 | 5.05 | 3.51 |
| PPARG | 4.82 | 3.64 | 4.68 | 5.71 | 4.43 |
